# Development and application of a high throughput carbohydrate profiling technique for analyzing plant cell wall polysaccharides and carbohydrate active enzymes

**DOI:** 10.1186/1754-6834-6-94

**Published:** 2013-07-03

**Authors:** Xiaofei Li, Peter Jackson, Denis V Rubtsov, Nuno Faria-Blanc, Jenny C Mortimer, Simon R Turner, Kristian B Krogh, Katja S Johansen, Paul Dupree

**Affiliations:** 1 Department of Biochemistry, Building O, Downing Site, University of Cambridge, Cambridge CB2 1QW, UK; 2 Faculty of Life Sciences, Michael Smith Building, Oxford Road, Manchester M13 9PT, UK; 3 Novozymes A/S, Krogshoejvej 36, Bagsvaerd 2880, Denmark

**Keywords:** DNA-sequencer, Cell wall, Glycosyl hydrolases, Glycosyl transferases

## Abstract

**Background:**

Plant cell wall polysaccharide composition varies substantially between species, organs and genotypes. Knowledge of the structure and composition of these polysaccharides, accompanied by a suite of well characterised glycosyl hydrolases will be important for the success of lignocellulosic biofuels. Current methods used to characterise enzymatically released plant oligosaccharides are relatively slow.

**Results:**

A method and software was developed allowing the use of a DNA sequencer to profile oligosaccharides derived from plant cell wall polysaccharides (DNA sequencer-Assisted Saccharide analysis in High throughput, DASH). An ABI 3730xl, which can analyse 96 samples simultaneously by capillary electrophoresis, was used to separate fluorophore derivatised reducing mono- and oligo-saccharides from plant cell walls. Using electrophoresis mobility markers, oligosaccharide mobilities were standardised between experiments to enable reproducible oligosaccharide identification. These mobility markers can be flexibly designed to span the mobilities of oligosaccharides under investigation, and they have a fluorescence emission that is distinct from that of the saccharide labelling. Methods for relative and absolute quantitation of oligosaccharides are described. Analysis of a large number of samples is facilitated by the DASHboard software which was developed in parallel. Use of this method was exemplified by comparing xylan structure and content in *Arabidopsis thaliana* mutants affected in xylan synthesis. The product profiles of specific xylanases were also compared in order to identify enzymes with unusual oligosaccharide products.

**Conclusions:**

The DASH method and DASHboard software can be used to carry out large-scale analyses of the compositional variation of plant cell walls and biomass, to compare plants with mutations in plant cell wall synthesis pathways, and to characterise novel carbohydrate active enzymes.

## Background

The plant cell wall is a complex structure, consisting of polysaccharides, lignin and protein, and forms the majority of biomass on the planet. Enzymatic saccharification of cell wall polysaccharides (lignocellulosic biomass) is likely to be an excellent source of sustainable energy, particularly when fermented to produce liquid biofuels [1]. A major challenge for producing biofuels cost-effectively is the efficiency of hydrolysis of polysaccharides to fermentable sugars. This requires knowledge both of the composition and structure of the biomass, and also the manufacture of effective glycosyl hydrolase (GH) cocktails.

The major polysaccharide of the cell wall is the semi-crystalline glucan, cellulose. In addition, there are hemicelluloses (such as xylan and xyloglucan) and pectins (e.g. polygalacturonan and rhamnogalacturonan I) which coat the cellulose fibrils. There is large variability in the structure of these polymers and in the composition of biomass from different plant organs, genotypes, species and between crops grown at different times [2], and therefore it will be important to be able to study the composition of biomass and the sugars released by GH cocktails in relatively high throughput and with simple and robust equipment.

Currently, many GH enzymes are assessed for their saccharification efficiency by colourimetric assays that quantify total sugars released, such as the dinitrosalicylic acid (DNS) assay, or 3-methyl-2benzothiazolinonehydrazone (MBTH) [3,4]. These methods support a high-throughput (HT) assay but do not provide detailed information on the sugars released. Alternatively, the released sugars can be structurally characterised individually e.g. by liquid chromatography (LC) or mass spectrometry (MS), but these methods (as discussed below) are labour-intensive and unsuitable for screening a large number of samples [5].

Analysis of cell wall polysaccharides is also a challenging task due to their heterogeneity in monosaccharide composition, linkage and glycoside sequence. A variety of techniques have been applied to the problem. For example, Fourier-Transform Infrared analysis, FT-IR, to image intact cell walls, can identify broad architectural alterations in cell walls [6]. However, the disadvantage of this method is that it provides little detailed information about changes to the structure of individual polysaccharides. More commonly, sequence and structural information is obtained by extraction and separation of polymers and fragmentation of the polymers to oligosaccharides. Compositional information is obtained by complete hydrolysis of the polymers to monosaccharides [7]. However, the fractionating process is time consuming, and the resulting oligosaccharide fractions are still complex mixtures, often confounding analysis using NMR and MS.

Another approach is to use specific GHs to digest the plant polysaccharide of interest to produce oligosaccharides which retain enough structural linkage information to determine the polysaccharide fine structure. For example, high performance anion exchange chromatography with pulsed amperometric detection (HPAEC-PAD) has been applied successfully to separate oligosaccharides [8]. HPAEC-PAD uses high ionic eluents that make on-line MS identification of oligosaccharides difficult. In addition, HPAEC-PAD can quantify oligosaccharides only if standards are available. Hydrophobic Interaction (HILIC)-HPLC coupled with matrix-assisted laser desorption/ionization-time-of flight/time-of-flight (MALDI-TOF/TOF) tandem MS has successfully been used to elucidate the structure of isomeric arabinoxylan oligosaccharides [8,9]. However, whilst HILIC coupled with MALDI-TOF/TOF MS can characterize unknown oligosaccharides, it cannot provide quantitative information without isotopic labelling [10].

Some of these disadvantages were overcome by the development of the PACE (Polysaccharide Analysis by Carbohydrate gel Electrophoresis) method, in which the reducing end of plant polysaccharide-derived oligosaccharides (released by hydrolases or other carbohydrate active enzymes) are labelled with a fluorophore by reductive amination, and then separated by polyacrylamide gel electrophoresis [11]. The method is robust and simple requiring little specialist equipment, but PACE is a relatively low throughput method requiring considerable user time.

An additional electrophoretic analytical technique uses capillary electrophoresis with laser-induced fluorescence (CE-LIF). CE-LIF is a powerful technique offering high detection sensitivity and good resolving capacity for the analysis of 9-aminopyrene-1, 4, 6-trisulfonate (APTS) labelled carbohydrates [12-15]. The method provides a possibility for quantification of carbohydrates by adding internal standards. Although numerous researchers have developed and used the method e.g. [16-20], it has significant limitations in terms of throughput.

Transfer of CE-LIF to a DNA sequencer provides several possible advantages. First, samples can be analysed in parallel, and some models allow concurrent CE of 96 samples. Second, the instruments can be adjusted to detect up to 5 specific fluorescent emission wavelengths potentially allowing spectral separation of mobility markers, standards and sample oligosaccharides. Most studies have investigated application to glycans larger than five or more residues and have incorporated glycan clean-up steps of HILIC or size exclusion chromatography to remove noise from unincorporated APTS [21-23]. However, for analysis of plant biomass and carbohydrate active enzyme products, it is important to be able to study monosaccharides and small oligosaccharides. Moreover, little is known about the fluorophore labelling efficiency and mobility variations between separate capillaries of plant cell wall-derived oligosaccharides [21,24].

Here, we present a high throughput method for quantitative analysis of plant cell wall polysaccharides using a 96 capillary array DNA sequencer (ABI 3730xl). We call this method DNA sequencer-Assisted Saccharide analysis in High throughput (DASH). In order to demonstrate the use of DASH for comparison of polysaccharide structures in plant biomass samples, the xylan structure of *Arabidopsis* xylan synthesis mutants was analysed. Additionally, DASH was used to investigate the substrate specificity of GHs, and to classify them by their product profiles.

## Results and discussion

### Oligosaccharide separation using a capillary DNA sequencer

**Figure 1:**
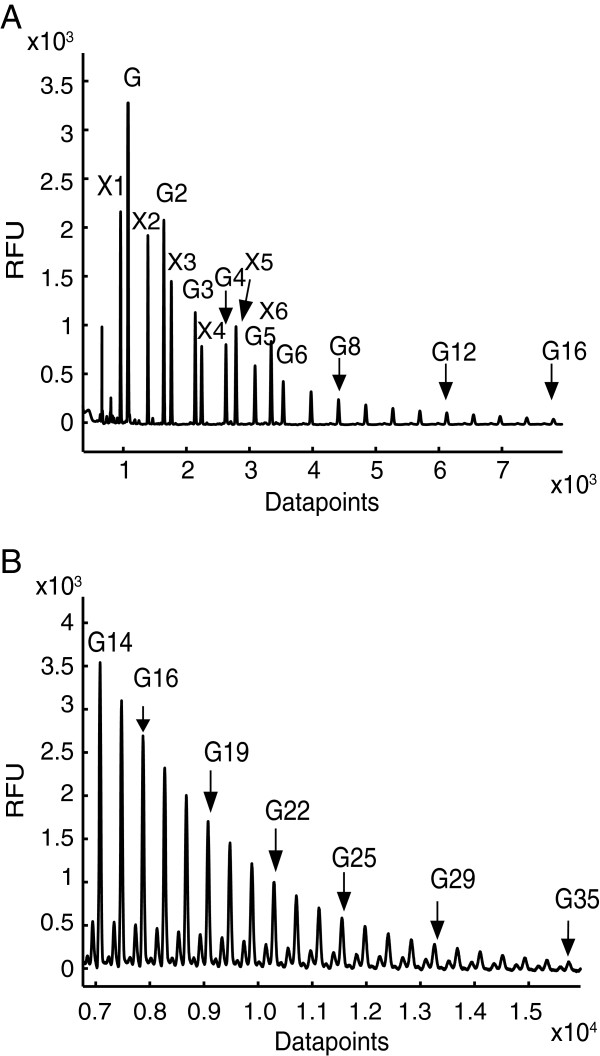
**Separation of APTS-labelled oligosaccharides by CE in an ABI 3730xl DNA sequencer. (A)** Electropherogram trace (50 min) of APTS labelled hydrolysed dextran and β-1,4-xylo oligosaccharides DP1 to DP6. **(B)** Large DP hydrolysed dextran oligosaccharides can be resolved by extending the electrophoresis time to 90 minutes. RFU, relative fluorescence units; G, Glucose; X, Xylose.

We first investigated the capability of CE-LIF in the ABI 3730xl DNA sequencer to resolve oligosaccharides prepared from plant cell wall polysaccharides. β-1,4 xylan oligosaccharides with degree of polymerization (DP) 1 to 6 were mixed with a dextran (α-1,6 glucan) ladder, and labelled with a single APTS at their reducing end. After dilution of samples to ~1 pmol in a 96-well microtitre plate, the samples were electrophoresed in parallel. The injection and running conditions were as described in Table 1. The APTS labelled oligosaccharides were detected in the blue channel of the DNA sequencer. As previously reported for separation of starch and protein N-glycans [21,25], the electropherogram traces demonstrated a good separation of xylan oligosaccharides (Figure 1A). Xylan oligomers were also well resolved from the dextran (Glc) oligomers. Oligosaccharides with higher DPs have lower electrophoretic mobility, but unlike liquid chromatography separations, they remain widely spaced (Figure 1A). In a 50 minute electrophoresis (8000 datapoints), the largest dextran oligosaccharide detected had a DP of 16. Dextran oligosaccharides with DP up to 35 were detected by using double the electrophoresis run time (Figure 1B), illustrating the flexibility of the method in targeting oligosaccharides with a wide range of sizes. The presence of nuisance peaks at the beginning of traces (up to approximately 900 datapoints) that arise due to unreacted APTS or other labelling reaction artefacts, do not interfere with monosaccharide or oligosaccharide peaks, the first of which appears at around 1000 datapoints (Figure 1A).

**Table 1:** Data acquisition settings for DASH

**Name**	**Value**
Oven temperature	30°C
Current stability	30 μA
Prerun voltage	15 kV
Prerun time	30 s
Injection voltage	2 kV
Injection time	20 s
Voltage number of steps	10
Voltage step interval	20 s
Data delay time	500 s
Run voltage	15 kV
Run time	50 (or 100) min
Ramp delay	1 s

### Oligosaccharide fluorescence quantitation and detection limits

**Figure 2:**
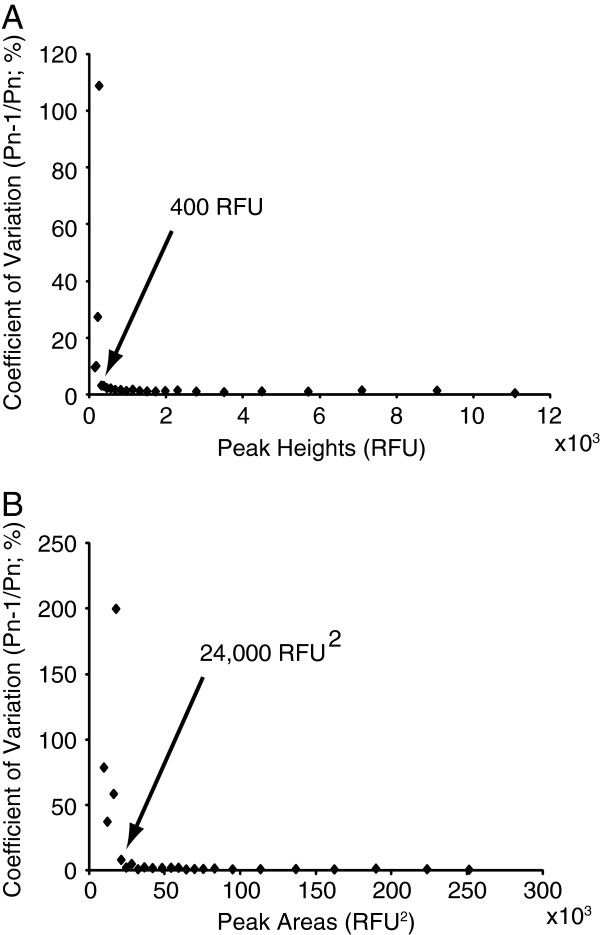
**Reproducibility of quantitation of oligosaccharides in parallel capillaries. (A)** Peak height measurements are highly reproducible. **(B)** Peak area measurements are highly reproducible. Below approximately height 400 RFU or area 20000 RFU^2^, the measurements are less reproducible.

The relative quantity of different APTS labelled oligosaccharides within a sample can be determined by the relative fluorescence intensity of the corresponding peaks, if the oligosaccharides are equally efficiently labelled with APTS. The fluorescence detection system has an upper limit of 35,000 relative fluorescence units (RFU) above which the response is saturated (data not shown). To determine the lower fluorescence unit limit for reproducible quantitation of oligosaccharides, a dextran ladder comprising a range of different quantities was analysed. To obtain comparable values between different CE traces, the peak area ratio and the peak height ratio between all adjacent DP peaks (P_n-1_/P_n_) were determined with DASHboard software (see methods). The variation in these ratios between 12 samples run in parallel was determined, and the coefficient of variation (CV) was calculated for oligosaccharides with a range of abundances (Figure 2). Peaks with a height over 400 RFU and peak area over 24,000 RFU^2^ were highly reproducible with a CV of less than 5%. Peaks with values lower than these resulted in lower reproducibility. Using standard oligosaccharides of known quantity, the quantity of oligosaccharide in the sample corresponding to the minimum RFU was determined to be approximately 50 fmol. The method is therefore highly sensitive. Moreover, below this limit of reproducible quantitation, the smallest detectable peak was about 50 RFU in height, corresponding to approximately 6 fmol.

### Linearity of saccharide derivatisation with APTS

APTS derivatisation must be proportional to oligosaccharide quantity in order to ensure reproducible and accurate quantitation. Carbohydrates with reducing ends could be completely derivatised with APTS through reductive amination. However, in practice the reaction may not go to completion, and the rate of labelling for different saccharides may vary, perhaps because different reducing carbohydrates have variations in the proportion or rate of interconversion between open versus closed ring-structures [26]. It has also been reported that insufficient APTS in the labelling reaction may cause preferential derivatisation of certain saccharides [27]. These issues may lead to different labelling efficiencies in a mixture of saccharides.

**Figure 3:**
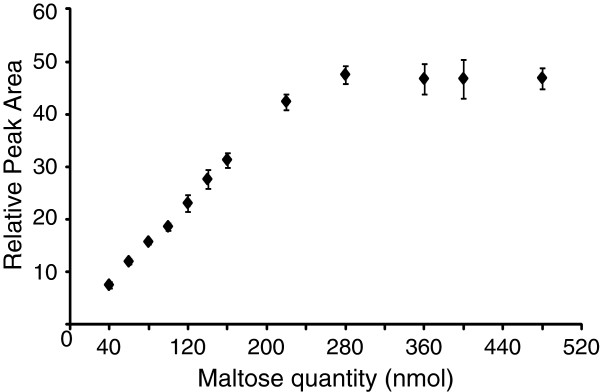
**Peak area is proportional to oligosaccharide quantity.** Maltose was derivatised with APTS and the resulting peak area compared to an internal standard. Bars represent s.d, n=3.

Therefore the linearity of saccharide derivatisation with the APTS fluorophore was investigated. Derivatisation protocols based on the method of Jackson [28] were optimised to ensure maximum saccharide labelling (data not shown), and the resulting labelling protocol is described in materials and methods. Figure 3 shows the relationship between maltose quantity and peak area (relative to a constant pre-labelled standard) . Keeping the APTS quantity constant at 200 nmol, with increasing sugar quantities in the labelling reaction, the peak area increased linearly until the sugar quantity reached about 200 nmol (*R*^*2*^ = 0.99). For greater oligosaccharide quantities, it is likely that saturation occurred due to the decrease in available APTS. Therefore, in the labelling protocol the quantity of all reducing sugars must be less than 200 nmol. Unknown amounts of reducing sugars can be determined relative to quantitation standards (QS) labelled together with these sugars. However, even in the presence of excess APTS, we found that labelled xylo-oligosaccharides gave more fluorescence than corresponding quantities of gluco-oligosaccharides (Additional file 1: Figure S1). Therefore the difference in labelling efficiency between sample and QS saccharides should be taken into account in quantitation calculations. Ideally, the QS should have the same reducing end saccharide as the analysed sugars to ensure equivalent labelling and accurate quantitation.

### Mobility markers can be used to align traces

The mobilities of APTS-labelled saccharides, when electrophoresed simultaneously in separate capillaries, show small but significant variations. Consequently, fluorescence peaks of a specific analyte may be detected at substantially different trace data points. This prevents confident identification of saccharides when using the peak data point. Moreover, in addition to variation in absolute elution time (shifts between traces), variation is also observed in the difference between elution times of pairs of analytes (for example through compression of regions of traces). In order to overcome these problems mobility markers (MMs) can be introduced to normalize the electropherogram traces. APTS-tagged glycan mobility markers that bracket the region of interest have been explored previously by various groups, but the traces are not aligned with sufficient accuracy for confident assignment of all analytes [15,29]. Trace alignment would be improved by employing markers throughout the region of interest, but there is potential co-migration of analytes with the APTS-tagged mobility marker sugars. To calibrate precisely oligosaccharide mobility data, we developed a new type of MM, using the ability of the ABI 3730xl system and similar DNA sequencers to detect multiple (usually five) colour dyes in a single capillary. Firstly, the MMs introduced here were labelled with the fluorophore DY-481XL, which has a fluorescence emission at a much longer wavelength (650 nm) than APTS (512 nm). The fluorescence of both can be easily resolved spectrally by the DNA sequencer and ensures effectively no detection of either fluorophore in the detection channel of the other fluorophore. Secondly, the fluorophore is conjugated to amino acids or short peptides that have different migration times. Targeted selection of different peptides allows the position of the MMs to be tuned to span the migration of the oligosaccharide analytes under study.

**Figure 4:**
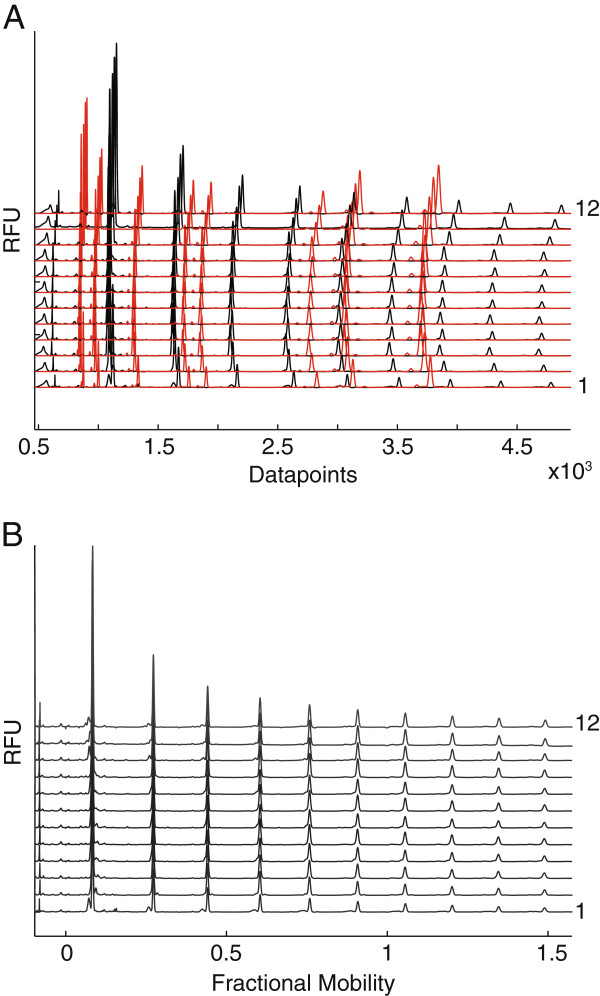
**Normalization of oligosaccharide mobility using internal markers with spectral properties distinct from APTS-labelled oligosaccharides. (A)** Dextran ladder (black) and mobility markers (red) reveal variation in electrophoresis between capillaries. **(B)** Electropherograms of dextran oligosaccharides after alignment of the mobility markers.

To investigate the accuracy of trace alignment, eight MMs labelled with DY-481XL were selected to span dextran oligosaccharides DP1 to DP6. These were added to each APTS labelled glycan sample prior to electrophoresis. The MMs and APTS-labelled sugars were monitored in two different channels, shown with red and black colours respectively in Figure 4A. The eight MMs have a range of different mobilities allowing accurate alignment along the entire saccharide profile. Alignment of oligosaccharide traces was carried out with the DASHboard software. After alignment, the oligosaccharide mobilities are expressed as a fractional mobility value, where zero is the mobility of the fastest MM and one is the mobility of the slowest MM. The alignment of the traces substantially reduced the variations in oligosaccharide mobility in parallel capillaries (Figure 4B). The fractional mobility of some oligosaccharides is shown in Additional file 2: Table S1.

### Polysaccharide analysis of *Arabidopsis* cell walls

Enzymatic digestion is a useful tool for elucidation of structural information and for quantitating polysaccharides and oligosaccharides [11]. To investigate whether DASH could allow reliable quantitation of polysaccharides in complex mixtures derived from the plant cell wall, we analysed the structure and quantity of xylan in *Arabidopsis* xylan synthesis mutants [30]. A number of proteins have been identified as putative glycosyltransferases (GTs) in xylan synthesis. IRX9 and IRX14 are involved in xylan backbone synthesis [30,31]. IRX7, IRX8 and PARVUS are required, in an unknown manner, for synthesis of a short xylan reducing end oligosaccharide (REO) [30-32].

Cell wall (100 μg) was pre-treated with alkali to make the xylan more accessible to hydrolytic enzymes. The enzyme-accessible xylan was then completely digested by adding excess GH family 10 (GH10) β-xylanase (*Cj*Xyn10A), and the released xylo-oligosaccharides quantifed by DASH, using QS. The total reducing sugar quantity in each sample was below 200 nmol, to ensure reproducible derivatisation as described above.

**Figure 5:**
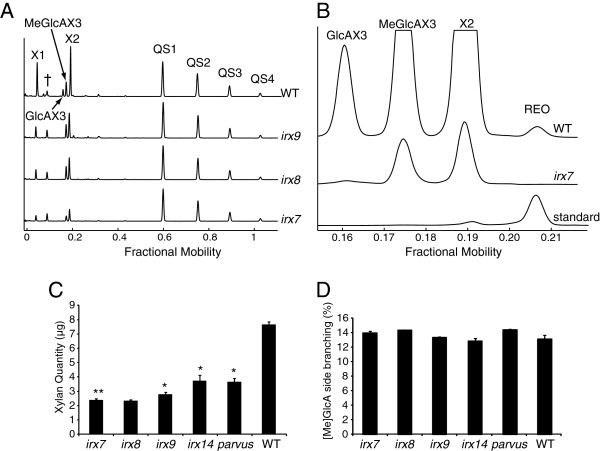
**Analysis of xylan structure and quantity by DASH.** Stem cell wall xylan of Wild type (WT) and mutant *Arabidopsis irx7*, *irx8*, *irx9, irx14* and *parvus* was hydrolysed with xylanase *Cj*Xyn10A, and the resulting oligosaccharides mixed with quantitation standards QS1-QS4. **(A)** Aligned electropherograms showing *Cj*Xyn10A digests. † denotes background peaks. **(B)** Identification of xylan reducing end oligosaccharide (REO) in WT is confirmed by co-migration with a standard, and absence in *irx7*. **(C)** Amount of xylan and **(D)** Percentage of [Me]GlcA branching of xylan in WT and *irx* mutants, quantified from *Cj*Xyn10A digests, and analysed using DASHboard. Data are the average of three technical replicates, and are shown +/- s.d. Values significantly different from WT are marked with a * (Student’s *t*-test, p < 0.05).

Figure 5A shows the profile of wild-type (WT) and three *irx* mutants. The traces of digested WT xylan revealed four abundant enzyme-specific peaks. The products of the GH10 enzyme digestion of *Arabidopsis* glucuronoxylan are well known [30]. The peaks on DASH were therefore easily identified as Xyl, (Xyl)_2_, glucuronic acid (GlcA) (Xyl)_3_ and 4-*O*-methyl glucuronic acid (MeGlcA) (Xyl)_3_. There was relatively little background in the region of Xyl, (Xyl)_2_ allowing analysis of monosaccharides and small oligosaccharides without sample clean-up_._ In contrast to PACE, it was possible to resolve GlcA(Xyl)_3_ and MeGlcA(Xyl)_3_ oligosaccharides (together named [Me]GlcA(Xyl)_3_). This is noteworthy as the two structures only differ in the presence of a 4-*O*-methyl ether leading to a size difference of only 14 Da. The *Cj*Xyn10A fingerprint of the *irx* mutants show only three major products: Xyl, (Xyl)_2_ and MeGlcA(Xyl)_3_. Unmethylated GlcA is absent, which is consistent with previous data from MS [30]_._ Also consistent with other studies, peak heights and areas are reduced in the mutants compared to WT suggesting lower levels of xylan [30,31]. In addition, a low abundance oligosaccharide can be detected migrating at fractional mobility 0.207. Co-migration with a standard showed the oligosaccharide to be the REO of xylan (Figure 5B). Consistent with previous data, this oligosaccharide was absent in the three mutants *irx7*, *irx8* and *parvus* (Figure 5B and data not shown), [30,31]. The ability to detect the REO, which is present at one copy per xylan polysaccharide molecule, demonstrates the sensitivity of the DASH technique.

The absolute quantity of xylan in five mutants was calculated by using internal quantitative standards (Figure 5C). As expected, all mutants show a large decrease in xylan quantity compared to the WT. The [Me]GlcA branching frequency was calculated and found not to differ significantly between the mutants and WT plants (Figure 5D), again consistent with previous reports [30].

**Figure 6:**
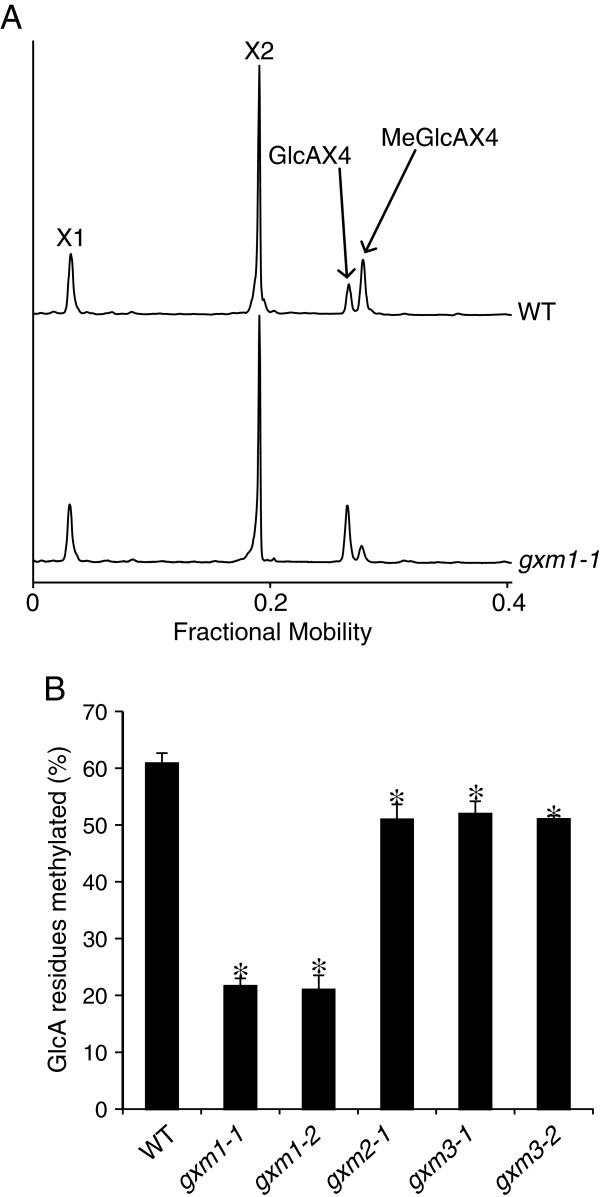
**Identification of xylan glucuronoxylan O-methyltransferases (GXM) by DASH.** Stem cell wall xylan of Wild type (WT) and *gxm* mutants was hydrolysed with *Np*Xyn11A. **(A)** Aligned electropherograms of *Np*Xyn11A digests of WT and *gxm1* AIR show altered proportions of methylated and unmethylated GlcAX_4_. **(B)** Proportion of MeGlcA substitution of xylan was quantified by integration of peak area and analysed using DASHboard. Data are the average of three biological replicates, and are shown +/- s.d. Values significantly different from WT are marked with a * (Student’s *t*-test, p < 0.05).

To further illustrate the power of DASH to resolve closely migrating oligosaccharides, we characterised further mutants in three closely related proteins in the DUF579 family. We previously found some members of this putative methyltransferase family to be localised in the Golgi apparatus [33]. Three DUF579 proteins, related to but distinct from IRX15 and IRX15L, are co-expressed with xylan synthesis enzymes [33,34]. One of these, GXM1, was recently shown to be a xylan GlcA methyltransferase [35]. The structure of xylan in this mutant was investigated by DASH using excess GH family 11 (GH11) β-xylanase (*Np*Xyl11A). As shown in Figure 6A, the digestion profile of WT and *gxm1* traces presents Xyl, (Xyl)_2_, GlcA(Xyl)_4_ and MeGlcA(Xyl)_4_. As expected, the proportions of GlcA(Xyl)_4_ and MeGlcA(Xyl)_4_ were significantly altered in *gxm1* compared to WT. To investigate whether GlcA methylation is dependent on additional DUF579 proteins, the structure of enzyme-accessible xylan was investigated in mutants of the three DUF579 proteins *gxm1*, *gxm2* and *gxm3*. Using peak areas of the GH11-released substituted xylan oligosaccharides, the percentage of methylated GlcA residues was calculated in two independent alleles for each mutant (Figure 6B; we were unable to obtain a second null allele for *gxm2*). All mutants showed decreased methylation in comparison to WT, which has 61% of GlcA methylated. *gxm1* has a reduction of methylation to 21% of GlcA (a reduction of 65% compared to WT), which is similar to previously obtained data by ^1^H NMR spectroscopy [35]. *gxm2* and *gxm3* each have a small but significant decrease in methylated GlcA to 51% (a reduction of 16% compared to WT). These figures are consistent with a recent examination of one allele of the mutants by NMR [36] and confirmed the reproducible and quantitative nature of the DASH analysis.

### Screening novel hydrolytic enzymes

**Figure 7:**
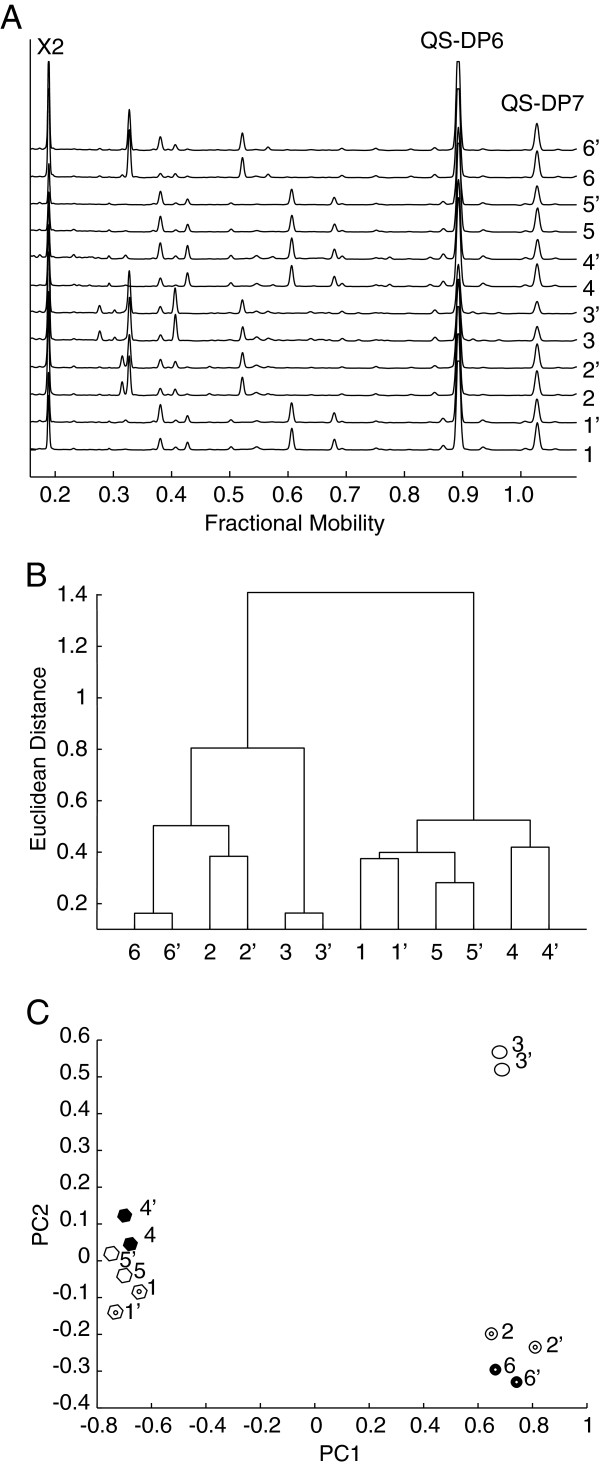
**Quantitative analysis of oligosaccharides released by wheat arabinoxylan hydrolysis with different xylanases. (A)** Aligned electropherograms of duplicate hydrolysis with xylanase 1 to xylanase 6. QS3 and QS4 are annotated. **(B)** Hierarchical clustering of quantities of oligosaccharides released by enzymes 1 to 6 using standardized Euclidean distances and DASHboard. **(C)** Principal components analysis of quantities of oligosaccharides released by xylanases 1 to 6.

Fingerprinting of polysaccharide hydrolase product profiles may reveal differences in enzyme activity. We investigated whether the technique could reveal product differences between related xylanase enzymes. Figure 7A shows aligned traces of duplicate digests of wheat flour arabinoxylan (WAX) with six different hydrolytic enzymes. The traces show that the enzymes released several arabinoxylan oligosaccharides. The oligosaccharides were quantified using two QS (DP6, DP7). The enzyme products were grouped by hierarchical clustering of these oligosaccharide abundances (Figure 7B). Pairs of duplicate digests clustered together. Based on the pattern of oligosaccharides released after digestion, the hydrolytic enzymes were clearly separated in two different groups. These groups correspond to enzymes from CAZy family GH10 (Enzymes 2, 3, 6) and GH11 (Enzymes 1, 4, 5). Principal component analysis (PCA) of the same data also resolved these two groups along PC1 (Figure 7C). However, PC2 additionally separated GH10 enzyme 3 from the other GH10 enzymes, indicating that the enzyme produced different oligosaccharide products.

## Conclusion

DASH profiling of plant cell wall polysaccharides is a fast method to detect and quantify oligosaccharides released from plant biomass by hydrolytic enzymes. DASH can provide absolute quantitation of products and information on the identity of oligosaccharides. The sensitivity of DASH is at the fmol level, requiring only a few μg of cell wall material, making microanalysis of different plant tissues feasible. A drawback of oligosaccharide mobility variation from capillary to capillary and from run to run has been resolved by using adjustible mobility markers. The markers developed here enable the standardisation of mobility from different experiments and equipment, without interfering with APTS-labelled sugar mobilities or their detection. Identification of peaks from aligned DASH traces is more accurate, which is essential for identification of peaks which migrate closely together. The tailor-made software package DASHboard ensures quick and flexible analysis of the data generated.

Validation of DASH as a method for analysing plant cell wall mutants was performed using a set of known xylan synthesis mutants, and the results were consistent with published data. DASH was then used to quantify the importance of methyltransferases that methylate GlcA on xylan. This was only possible due to the high reproducibility in separation and migration of GlcA and MeGlcA substituted xylo-oligosaccharides by DASH using the MMs. DASH was also validated as a high-throughput method for characterisation and classification of enzymes based on their products, here shown for different xylanases. All in all, DASH in combination with the DASHboard data analysis allows the identification of differences in oligosaccharide patterns derived from different cell wall oligosaccharides and products of diverse hydrolases to be analysed in high throughput with high reproducibility.

Identification of unknown oligosaccharide peaks could involve comigration studies with standards or determination of oligosaccharide sensitivity to diagnostic glycosidases. In the future, a DASH oligosaccharide mobility library will be established, to assist in the automatic identification of oligosaccharides from plant biomass. Data will be transferrable between research groups, due to the consistency in mobility provided by the MMs.

## Methods

### Reagents

Endo-β-1,4-xylanases in CAZy family GH10 (*Cj*Xyn10A from *Cellvibrio japonicus* and *Cm*Xyn10A from *Cellvibrio mixtus*) and GH 11 (*Np*Xyn11A from *Neocallimastix patriciarum* and *Ev*Xyn11 from an uncultured bacterium) were a generous gift from Professor Harry Gilbert and Dr David Bolam (University of Newcastle, Newcastle-upon-Tyne, UK). Endo-β-1,4-xylanases NZGH10 and NZGH11 were a generous donation from Novozymes A/S (Bagsvaerd, Denmark). 8-Aminopyrene-1,3,6-trisulfonic acid, trisodium salt (APTS) was supplied by (Biotium, Cambridge BioScience, Cambridge, UK). The malto-oligosaccharides DP4-7 (maltotetraose (Glc-4), maltopentaose (Glc-5), maltohexaose (Glc-6) and maltoheptaose (Glc-7)), mannose, cellobiose, Dextran 10 and sodium cyanoborohydride (NaCNHB_3_) were supplied by Sigma-Aldrich Co. Ltd (Dorset, UK). The xylo-oligosaccharides (DP1-6) and wheat arabinoxylan (WAX) were supplied by Megazyme (Megazyme International Ireland, Bray, Ireland). Hi-Di™ formamide was supplied by Applied Biosystems (Life technologies, Paisley, UK). A set of 7 amino acids and peptides was selected for use as components of the electrophoretic mobility standards used in this study as follows, listed in order of decreasing electrophoretic mobility of their fluorophore derivatives; Asp-Asp-Asp-Asp (Bachem AG, Bubendorf, Switzerland), Asp-Asp-Asp (Bachem), Glu-Glu (Sigma-Aldrich), Cysteic acid (Sigma-Aldrich), L-2-Aminoadipic acid (Sigma-Aldrich), Glycine (Sigma-Aldrich), Gly-Gly-Gly (Sigma-Aldrich). The fluorophore for the mobility standards, DY-481XL-NHS ester, was obtained from Dyomics Gmbh, Jena, Germany.

### Plant material

*Arabidopsis thaliana* material used was as follows: wild type (WT; ecotype Col0); *irx7*, *irx8*, *irx9*, *irx14,* and *parvus* as described in [30]; *gxm1-1* (SALK_018081), *gxm2-1* (SALK_084669) and *gxm3-1* (SALK_050883) as described in [34]. Seeds of T-DNA insertion lines GK-677C12_023105 (*gxm3-2*) and SALK_087114 (*gxm1-2*) were obtained from the Nottingham Arabidopsis Stock Centre, homozygous lines isolated, and confirmed as null mutants as described in [30]. Plants were grown on soil (Levington M3) in a growth room (21°C, 100 μmol m^-2^ s^-1^, 16 h light/8 h dark, 60% humidity). Basal stem material was harvested once the stem had at least 10 siliques (6–7 weeks after germination) andwere incubated in 96% (v/v) ethanol (70°C, 30 mins) to inactivate enzymes.

The stems were ground to a homogenised powder using a ball mixer mill (Glen Creston Ltd, Midddlesex, UK), and used to prepare alcohol insoluble residue (AIR). The solid matter was collected by centrifugation (10 mins, 4,000 x g), and washed using the following steps: 100% (v/v) ethanol, twice with chloroform:methanol (2:1 (v/v)), followed by successive washes with 65% (v/v), 80% (v/v) and 100% (v/v) aqueous ethanol. The pellet was then air dried at 40°C overnight.

### Enzymatic hydrolysis of the cell wall extract for DASH fingerprinting

AIR (100 μg) was treated with 4 M NaOH (20 μL) for 1 h at room temperature (approx. 22°C) before adjusting the pH to pH 5–6 with 1 M HCl. Enzyme digests were performed in 500 μl 0.1 M ammonium acetate pH 6.0, and enzyme quantity was adjusted to ensure complete hydrolysis. The mixtures were boiled for 30 min to stop the reaction.

### Quantitative standards

The QS were prepared by combining maltotetraose (QS1), maltopentaose (QS2), maltohexaose (QS3) and maltoheptaose (QS4) in the molar ratios, 20:15:8:3. A total of 46 nmol of the 4 saccharides were added, in aqueous solution to the sample (e.g. enzyme digests). The mixtures were dried using a centrifugal vacuum evaporator CVE prior to labelling with APTS.

### Mobility markers

The mobility standards were prepared from an initial set of 26 labelled amines, either amino acids or peptides that were each derivatised separately through their primary amino groups with the fluorophore DY-481XL-NHS ester (Additional file 3: Figure S2). Each amine served to modulate the electrophoretic mobility of each fluorescent derivative of which it comprised. The resulting fluorescent derivatives had net charges ranging from -1 to -5 and molecular weight from 75.07 to 478.37. From these, seven standards (see Reagents) were selected to provide a set with a range of mobilities that extended from faster-than to slower-than any of the APTS-labelled saccharides analysed in this work. One additional mobility marker was not comprised by an amino acid or peptide but was a by-product of the labelling reaction. Other amines can be used to prepare mobility standards suitable for other investigations.

A 10 mM solution of each amine was prepared in 0.1 M NaHCO_3_. A 0.4 mM solution of the fluorophore DY-481xl-NHS ester was freshly prepared in dimethylsulfoxide. The mixture of DY-481xl-NHS ester (2.5 μl) and each amine (2.5 μl) was incubated for 60 min at room temp (22°C) in the dark and shaken vigorously for 10 sec every 5 min. Unreacted amines in the mixture were not detected by the DNA analyser. Each amine was labelled in a separate reaction. The reaction mixtures were each diluted with 0.5 mL water and stored as stock solutions at -20°C. The final working solution was a dilution of 10 μl of the stock with 390 μL water.

The samples were tested individually in the DNA sequencer to determine the peak heights by drying 2 μL and 5 μL final working solution from each in a 96-well microtitre plate using a CVE. The samples were dissolved in 20 μL formamide and analysed using the red channel of the DNA sequencer. The peak profiles for each of the selected standards was examined and the individual concentrated standards solutions mixed in appropriate amounts so that the peak heights of all the standards were approximately the same. The same volume of the mix of standards was added to each sample.

### APTS labelling and analysis of the labelled saccharides

A solution of APTS was prepared at a concentration 0.02 M in 1.2 M citric acid solution and stored at -20°C. A fresh solution of 0.1 M NaCNBH_3_ was prepared in water. The dried QS and oligosaccharide sample mixture were combined with 10 μL APTS and 10 μL NaCNBH_3_ solutions. Samples were mixed well, centrifuged briefly to bring the reaction mix to the tip of the tube and incubated in a (CVE) at 37°C for 6 h without vacuum.

The labelling reaction mixtures were diluted with water: for a reaction containing 100 nmol of total saccharide a dilution of 10^4^-fold was typical. Multiple different dilutions were tested to ensure that low abundance saccharides were detectable. Typically, a volume containing approximately 1 pmol of treated saccharide was placed in a well of a 96-well microtitre plate suitable for use with a DNA sequencer. The electrophoresis mobility marker solution (10 μL; preparation is described below) was added to each sample in the 96-well plate and dried in a CVE. Hi-Di™ formamide (20 μL, Invitrogen) was added to each sample well and the plate was shaken vigorously for 1 h to solubilise the labelled saccharides. The plate was used directly for sample analysis in an ABI 3730xl 96-sample DNA sequencer using the standard DNA analysis buffer system, and the settings described in Table 1. If necessary the fluorescence detection system can be calibrated to ensure that no fluorescence from the mobility standards (red channel) is detected in the blue channel used for the detection of the APTS-labelled saccharides and vice-versa. With standard DNA detection parameters there was no cross-over between the red and the blue channels.

### Screening hydrolytic enzymes

WAX (30 μg) was incubated overnight with the endo-β-1,4-xylanases in 500 μl 0.1 M ammonium acetate pH 6.0. The digestions were stopped by boiling for 30 minutes, and a reduced set of QS added (QS6 and QS7 only, due to co-migration of QS4 and QS5 with xylanase-digestion products of WAX). Following drying by CVE, the samples were derivatised with APTS, diluted and analysed using an ABI 3730xl DNA sequencer.

### Data analysis and the DASHboard software

DASH data generated by the DNA sequencer (.fsa file format) were processed in DASHboard software, which was written and developed using MATLAB (Mathworks Inc). The DASHboard software tool is designed to complement the profiling technique and perform essential data processing tasks such as reading the results of the scan, visualisation, peak quantification and export to Excel (.xls format) for further analysis. DASH files were loaded into DASHboard for data visualisation of the fluorescence peak profiles of the CE separations. DASHboard was used to align the peak profiles using the mobility standards that were present in every sample. Therefore, the profiles of the APTS-labelled saccharides in any sample could be overlaid exactly.

Quantitation of APTS-labelled saccharides was carried out using internal quantitative standards and analysis with the DASHboard software. A standard curve of peak areas versus the corresponding molar quantity of each APTS-labelled QS was used to determine the quantity of each APTS-labelled oligosaccharide.

For example, to calculate the *Arabidopsis* xylan quantity from a GH10 xylanase digestion, where the identity of all oligosaccharides is known, it was assumed that the xylanase digestions had gone to completion. Therefore, all the xylan was present as Xyl, (Xyl)_2_, GlcA(Xyl)_3_ and MeGlcA(Xyl)_3_. Xylose present in the digested xylan was the sum of the quantity of each of these digestion products multiplied by the number of xylose residues present in each structure: total Xyl = ((Xyl)_1_ x 1 + (Xyl)_2_ x 2 + GlcA(Xyl)_3_ x 3 + MeGlcA(Xyl)_3_ x 3).

Statistical analysis was also performed in DASHboard. Hierarchical clustering and PCA approaches were used to analyse rapidly cell wall data for classification and characterisation.

The DASHboard software tool will be made available on request.

## Abbreviations

AIR: Alcohol insoluble residue; APTS: 8-aminopyrene-1, 4, 6-trisulfonate; CAZy: Carbohydrate active enzyme database; CE-LIF: Capillary electrophoresis with laser-induced fluorescence; CVE: Centrifugal vacuum evaporator; DASH: DNA sequencer-Assisted Saccharide analysis in High throughput; DP: Degree of polymerization; DNS: Dinitrosalicyclic acid; FT-IR: Fourier-Transform Infrared analysis; GH: Glycosyl hydrolase; Glc: Glucose; GlcA: Glucuronic acid; HPAEC-PAD: High performance anion exchange chromatography with pulsed amperometric detection; HT: High-throughput; IRX: Irregular xylem; LC: Liquid chromatography; MALDI-TOF/TOF: Matrix-assisted laser desorption/ionization-time-of flight/time-of-flight; MBTH: 3-methyl-2benzothiazolinonehydrazone; MeGlcA: 4-O-methyl glucuronic acid; MM: Mobility markers; MS: Mass spectrometry; NP-HPLC: Normal phase-HPLC; PACE: Polysaccharide Analysis using carbohydrate gel electrophoresis; PCA: Principal component analysis; QS: Quantitation standards; RFU: Relative fluorescence units; WAX: Wheat arabinoxylan; WT: Wild type; Xyl: Xylose.

## Competing interests

A patent has been filed on the mobility markers. The authors declare no other competing interests.

## Authors’ contributions

XL and PJ carried out the method optimisation and prepared most of the data. DVR wrote the DASHboard software. NFB identified and analysed the GXM mutant plants. JCM contributed to method optimisation. ST contributed the IRX and GXM mutant candidates. PD, KSJ and KBK co-supervised the work. XL, JCM, PJ and PD wrote the paper. All authors read and approved the final manuscript.

## Supplementary Material

Additional file 1: Figure S1 Labelling efficiency of various monosaccharide and oligosaccharides. 100 nmol of each saccharide was labelled with APTS and the fluorescence detected of the labelled saccharides is expressed relative to glucose. n=3, ±SD. Click here for file

Additional file 2: Table S1 Fractional mobilities (FM) of some common monosaccharides and oligosaccharides used in the study of plant secondary cell walls. The samples are as follows: G1-G12 – acid hydrolysed dextran (α1,6-linked Glc), X1-6 - (β1,4-linked Xyl), M1-6 - (β1,4-linked Man). Xylo-oligosaccharides carrying α1,2 [Me]GlcA are well-characterised products of glucuronoxylan digestion with xylanases in CAZy families GH10 and GH11. Click here for file

Additional file 3: Figure S2 Structure of DY-481XL fluorophore label of MMs. Click here for file
