# Supplementary figures and images for: Development and application of a high throughput carbohydrate profiling technique for analyzing plant cell wall polysaccharides and carbohydrate active enzymes
Source: Biotechnol Biofuels. 2013 Jul 3;6:94. doi: 10.1186/1754-6834-6-94 (PMC3717103; doi:10.1186/1754-6834-6-94)

Fig S1

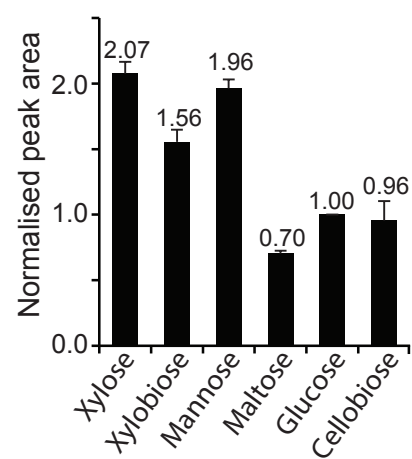

Supplement: Additional file 1: Figure S1 — Labelling efficiency of various monosaccharide and oligosaccharides. 100 nmol of each saccharide was labelled with APTS and the fluorescence detected of the labelled saccharides is expressed relative to glucose. n=3, ±SD. [file 1754-6834-6-94-S1.pdf]

Figure S2

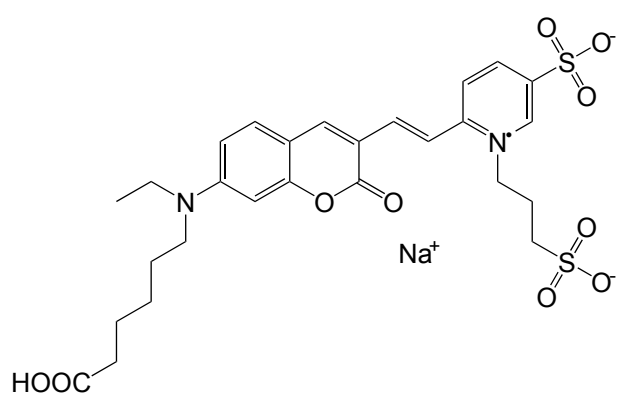

Supplement: Additional file 3: Figure S2 — Structure of DY-481XL fluorophore label of MMs. [file 1754-6834-6-94-S3.pdf]
