# Supplementary material for: Development and application of a high throughput carbohydrate profiling technique for analyzing plant cell wall polysaccharides and carbohydrate active enzymes
Source: Biotechnol Biofuels. 2013 Jul 3;6:94. doi: 10.1186/1754-6834-6-94 (PMC3717103; doi:10.1186/1754-6834-6-94)
Supplement: Additional file 2: Table S1 — Fractional mobilities (FM) of some common monosaccharides and oligosaccharides used in the study of plant secondary cell walls. The samples are as follows: G1-G12 – acid hydrolysed dextran (α1,6-linked Glc), X1-6 - (β1,4-linked Xyl), M1-6 - (β1,4-linked Man). Xylo-oligosaccharides carrying α1,2 [Me]GlcA are well-characterised products of glucuronoxylan digestion with xylanases in CAZy families GH10 and GH11. [file 1754-6834-6-94-S2.docx]

| **Peak Identity** | **FM** |
| --- | --- |
|  | |
| G1 | 0.096 |
| G2 | 0.283 |
| G3 | 0.451 |
| G4 | 0.612 |
| G5 | 0.765 |
| G6 | 0.913 |
| G7 | 1.059 |
| G8 | 1.204 |
| G9 | 1.347 |
| G10 | 1.489 |
| G11 | 1.630 |
| G12 | 1.770 |
|  | |
| Xylose | 0.042 |
| X2 | 0.188 |
| X3 | 0.313 |
| X4 | 0.475 |
| X5 | 0.658 |
| X6 | 0.845 |
|  | |
| GlcAX3 | 0.161 |
| MeGlcAX3 | 0.176 |
| GlcAX4 | 0.271 |
| MeGlcAX4 | 0.283 |
|  | |
| Mannose | 0.106 |
| M2 | 0.262 |
| M3 | 0.427 |
| M4 | 0.617 |
| M5 | 0.814 |
| M6 | 1.016 |

**Table S1.** Fractional mobilities (FM) of some common monosaccharides and oligosaccharides used in the study of plant secondary cell walls. The samples are as follows: G1-G12 – acid hydrolysed dextran (α1,6-linked Glc), X1-6 - (β1,4-linked Xyl), M1-6 - (β1,4-linked Man). Xylo-oligosacchadrides carrying α1,2 [Me]GlcA are well-characterised products of glucuronoxylan digestion with xylanases in CAZy families GH10 and GH11.
